# Commentary: Induction of Dormancy in Hypoxic Human Papillomavirus-Positive Cancer Cells

**DOI:** 10.3389/fonc.2018.00077

**Published:** 2018-03-27

**Authors:** Anita Szalmás

**Affiliations:** Department of Medical Microbiology, Faculty of Medicine, University of Debrecen, Debrecen, Hungary

**Keywords:** human papillomavirus, signaling, tumorigenesis, immunotherapy, hypoxia

High-risk human papillomavirus (HR-HPV) infection-associated cancers of the cervix, anogenital tract, and the nasopharynx represent an important clinical problem globally (1, 2). Mucosal HPV infections are highly prevalent; however, only a very small subset of HR-HPV infections persists and a small fraction these persistent infections will, in fact, lead to cancer development (3). Moreover, HPV-induced carcinogenesis takes many years or decades to develop and is a multistep process that requires additional tumor-promoting factors for invasive cancer to occur (3). It is now accepted that the immune system has a key surveillance function that can prevent persistent HPV infections and can also control the neoplastic transformation and the progression of premalignant lesions to invasive carcinoma (4, 5). Currently used regimens for the treatment of HPV-associated cancers have remained unchanged for decades and are of limited value for the treatment of patients with advanced or recurrent disease (6). The available prophylactic vaccines can protect against infections by the most common HR-HPV types, but they are not able to eliminate or prevent the malignant progression of persistent HPV infections (5). Therefore, immune-based therapies are considered as promising novel approaches for the prevention and effective treatment of HPV-associated diseases.

The key viral factors that are involved in the induction and maintenance of the malignant phenotype of the host cell are the HR-HPV E6 and E7 proteins (7, 8). These early HPV oncoproteins do not possess enzymatic activity, but rather manipulate the host cell by associating with a plethora of cellular proteins (9, 10). The main biological function of these interactions is to hinder the differentiation of the infected epithelial cell and keep it in a replication-competent state to ensure HPV genome replication. However, during HPV-associated carcinogenesis, HR-HPV E6 and E7 can also contribute to all of the main phenotypic alterations of malignant cells that have been named as “hallmarks of cancer” (11).

Both E6 and E7 represent ideal targets for immunotherapy, as they are immunologically foreign antigens. Furthermore, they are always retained and constitutively expressed by HPV-positive cancer cells as growth is strongly dependent on sustained E6/E7 expression (“oncogene addiction”) that ensures the continuous inactivation of tumor suppressor pathways. E6/E7 levels cannot be downregulated as an immune-evasion mechanism, because their inhibition leads to induction of irreversible proliferative arrest (cellular senescence) (11) (Figure 1). Moreover, a recent large-scale genomic survey suggests that the conserved sequence of E7 oncoprotein is critical for cervical carcinogenesis (12). Nevertheless, efforts to induce tumor regression by targeting the E6 and/or E7 oncoproteins by using immunotherapy, such as different types of therapeutic vaccines, had limited success (6, 13).

**Figure 1:**
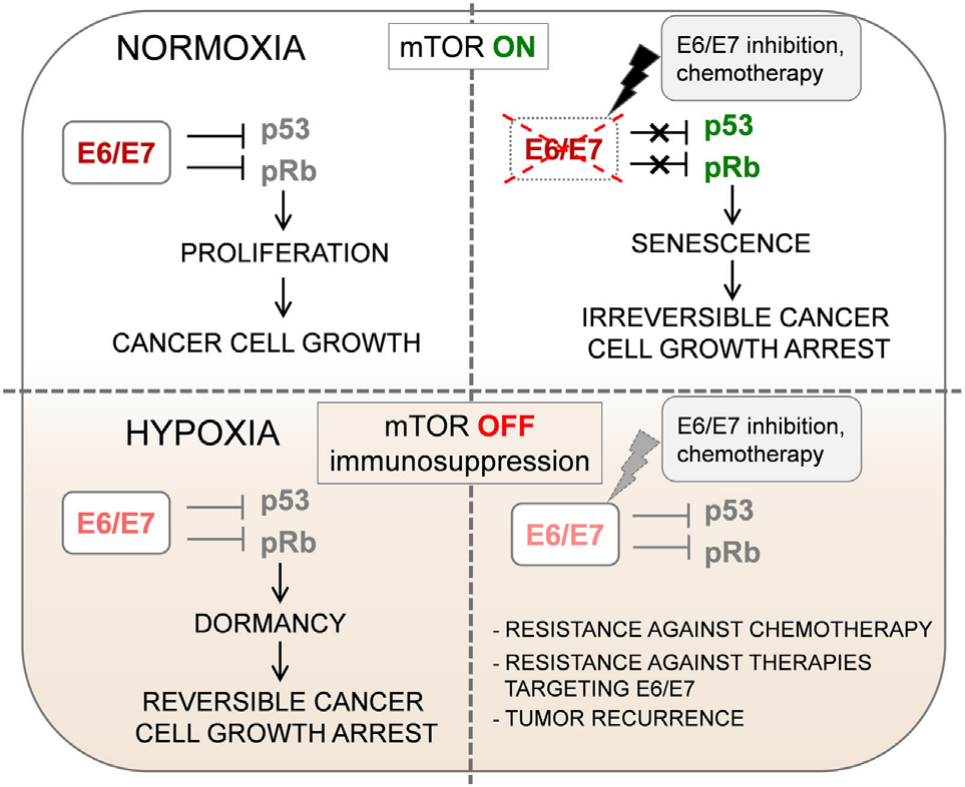
Oxygenation can change the biological behavior of HPV-positive tumors. Impaired mechanistic target of rapamycin (mTOR) signaling and concomitant HPV E6/E7 repression in hypoxic HPV-positive cancer cells allow the evasion of senescence. This has several clinical implications for the treatment of HPV-associated cancers: (1) dormant HPV-positive cancer cells in hypoxic subregions of tumors might lead to cancer recurrence if oxygen supply increases; (2) the growth inhibition under hypoxic conditions in cancer tissues results in increased therapeutic resistance against pro-senescent chemotherapeutic agents; (3) reduced expression of viral E6/E7 oncoproteins could represent a major obstacle for immunotherapy of HPV-associated cancers.

Recent evidence supports the importance of targeting non-viral antigens in immunotherapy of HPV-associated cervical cancer (14, 15). The continuous expression of HPV oncoproteins can cause genomic instability, which can subsequently lead to an increased somatic mutation rate and aberrations in the epigenetic regulation of tissue-specific gene expression. The altered protein products of somatic mutations (mutated neoantigens) and the aberrantly expressed epigenetically dysregulated genes (cancer germline antigens) may also elicit an effective immune response and, therefore, represent an additional immune-based therapeutic strategy against HPV-associated malignancies. A recent study demonstrated that in two patients with complete remission of HPV infection-associated metastatic cervical cancer after adoptive therapy with tumor infiltrating T lymphocytes (TILs), immunodominant T cell reactivities were directed against mutated neoantigens or a cancer germline antigen, rather than the HPV-specific antigens (16). This was observed in spite of the fact that the T cells used for adoptive therapy were selected for HPV antigen reactivity. Moreover, analysis of peripheral blood samples revealed that TILs against viral antigens did not show preferential *in vivo* expansion during cancer regression.

The above-mentioned article can provide a possible explanation for the limited efficacy of HPV antigen-specific immunotherapy and the lower than anticipated presence of HPV-specific TILs following adoptive immunotherapy (17). Hoppe-Seyler and colleagues made the remarkable observation that under hypoxic conditions that are often found in advanced stage cervical cancer as well as oral squamous cell carcinoma tissues, the expression of HPV E6/E7 viral oncoproteins is strongly downregulated. First, they observed that under hypoxia, HR HPV-positive cervical cancer cell lines show decreased expression of HPV E6/E7 oncogenes. They also observed a decreased E7 expression in hypoxic areas of HPV-16 positive cervical cancer tissue specimens. Interestingly, E6/E7 downregulation by hypoxia did not lead to the induction of cellular senescence in HPV-positive cervical cancer cells, instead, the cells underwent a reversible mitotic and growth arrest (dormancy) (Figure 1). Upon re-oxygenation, these dormant HPV-positive cells restored E6/E7 expression, which led to renewal of host cell proliferation.

They propose that the key metabolic pathway that ensures the induction of senescence upon E6/E7 downregulation under normoxia is the mechanistic target of rapamycin (mTOR) signaling pathway. Treatment of HPV-positive cancer cells with mTOR inhibitors released them from HPV oncoprotein “addiction,” thus, enabled the escape of cancer cells from senescence when E6/E7 expression is blocked. Next, they demonstrated that the lack of senescence induction observed in HPV-positive cancer cells cultured under hypoxic conditions is because of impaired mTOR signaling. In hypoxic HPV-18-positive HeLa cells, the hypoxia-inducible factor 1 downstream target regulated in development and DNA damage responses 1 (REDD1) was upregulated. REDD1 can interfere with mTOR signaling *via* activation of the mTOR inhibitor tuberous sclerosis complex 2 (TSC2); therefore, the authors propose that the REDD1/TSC2 axis might be responsible for the lack of senescence induction in hypoxic HeLa cells. Impairment of mTOR signaling by hypoxia (similarly to treatment with mTOR inhibitors) interfered with the senescence response of HPV-positive cancer cells against pro-senescent chemotherapeutic agents. The findings of this study demonstrate that the clinical outcome in patients with HPV-associated cancers can be strongly influenced by the metabolic state of the host cell (Figure 1). Thus, prospective pro-senescent E6/E7 inhibitors would be expected to have therapeutic potential in normoxic cancer cells with elevated E6/E7 expression and active mTOR signaling. However, to target HPV-positive cancer cells in hypoxic tumor sub-areas, viral oncoprotein inhibition could be combined with other approaches specifically targeting hypoxic cells/tissue regions or applying hypoxia-activated agents (18–21).

## Author Contributions

The author confirms being the sole contributor of this work and approved it for publication.

## Conflict of Interest Statement

The author declares that the research was conducted in the absence of any commercial or financial relationships that could be construed as a potential conflict of interest.
